# Frequencies of the *LILRA3* 6.7-kb Deletion Are Highly Differentiated Among Han Chinese Subpopulations and Involved in Ankylosing Spondylitis Predisposition

**DOI:** 10.3389/fgene.2019.00869

**Published:** 2019-09-18

**Authors:** Han Wang, Yuxuan Wang, Yundi Tang, Hua Ye, Xuewu Zhang, Gengmin Zhou, Jiyang Lv, Yongjiang Cai, Zhanguo Li, Jianping Guo, Qingwen Wang

**Affiliations:** ^1^Department of Rheumatism and Immunology, Peking University Shenzhen Hospital, Shenzhen, China; ^2^Department of Rheumatology and Immunology, Peking University People’s Hospital, Beijing, China; ^3^Health Management Center, Peking University Shenzhen Hospital, Shenzhen, China

**Keywords:** *LILRA3*, genetic differentiation, genetic susceptibility, ankylosing spondylitis, Han subpopulations

## Abstract

**Introduction:** Leukocyte immunoglobulin-like receptor A3 (*LILRA3*) belongs to the LILR family with unique feature of a 6.7-kb deletion variation among individuals. Frequencies of the 6.7-kb deletion vary widely across populations, but so far it has not been carefully investigated among Han Chinese subpopulations. Furthermore, we previously identified the non-deleted (functional) *LILRA3* as a novel genetic risk for multiple autoimmune diseases. The current study aimed to investigate (i) whether frequencies of the *LILRA3* 6.7-kb deletion differ within Han Chinese subpopulations and (ii) whether the functional *LILRA3* is a novel genetic risk for ankylosing spondylitis (AS).

**Methods:** The *LILRA3* 6.7-kb deletion was genotyped in two independent cohorts, including 1,567 subjects from Shenzhen Hospital and 2,507 subjects from People’s Hospital of Peking University. Frequencies of the 6.7-kb deletion were first investigated in combined healthy cohort according to the Chinese administrative district divisions. Association analyses were performed on whole dataset and subsets according to the geographic regions. Impact of the functional *LILRA3* on AS disease activity was evaluated.

**Results:** Frequencies of *LILRA3* 6.7-kb deletion were highly differentiated within Han Chinese subpopulations, being gradually decreased from Northeast (80.6%) to South (47.4%). Functional *LILRA3* seemed to be a strong genetic risk in susceptibility to AS under almost all the alternative genetic models, if the study subjects were not geographically stratified. However, stratification analysis revealed that the functional *LILRA3* was consistently associated with AS susceptibility mainly in Northern Han subgroup under the alternative genetic models, but not in Central and Southern Hans. Functional *LILRA3* conferred an increased disease activity in AS patients (*P* < 0.0001 both for CRP and ESR, and *P* = 0.003 for BASDAI).

**Conclusions:** The present study is the first to report that the frequencies of *LILRA3* 6.7-kb deletion vary among Chinese Hans across geographic regions. The functional *LILRA3* is associated with AS susceptibility mainly in Northern Han, but not in Central and Southern Han subgroups. Our finding provides new evidence that *LILRA3* is a common genetic risk for multiple autoimmune diseases and highlights the genetic differentiation among different ethnicities, even within the subpopulations of an ethnic group.

## Introduction

Ankylosing spondylitis (AS) is a chronic autoimmune disease characterized by new bone formation, progressively leading to ankylosis of the axial skeleton and functional disability. The disease predominantly affects young men. The etiology of AS is not completely understood, but it is believed that genetic factors play a major role in AS pathogenesis, particularly the MHC class I allele *HLA-B27*, which has been recognized as the best genetic marker for AS susceptibility (reviewed in (Brown et al., 2016)). However, despite over 80% of AS patients are *HLA-B27* carriers, only a small proportion of *HLA-B27* positive individuals ever develop AS (reviewed in (Reveille, 2012)). Furthermore, the genome-wide association studies (GWAS) have revealed that more than 60 additional genetic risk factors contributed to the disease, indicating a polygenic nature of AS. To date, only approximately 30% of AS heritability has been explained by the known genetic loci; many remain unidentified (reviewed in (Li and Brown, 2017; Ranganathan et al., 2017)).

The leukocyte immunoglobulin-like receptor genes (*LILRs*) is a highly homologous multigene family located on human chromosome 19q13.4 (Samaridis and Colonna, 1997; Kelley et al., 2005). One of characteristics of LILR family is their specific recognition of MHC class I molecules (Cosman et al., 1997). According to the signaling pathways through immunoreceptor tyrosine-based activating or inhibitory motifs, two subgroups of the LILRs have been defined: activating (LILRA1-6) and inhibitory (LILRB1-5) receptors (Samaridis and Colonna, 1997; Nakajima et al., 1999). Of which, LILRA3 (OMIM 604818) is unique, due to a premature stop codon in the extracellular stalk region, leading to a loss of transmembrane domain and therefore expressed only as a soluble receptor (Arm et al., 1997; Borges et al., 1997; Colonna et al., 1997). Furthermore, *LILRA3* exhibits a presence or absence of 6.7-kb variation among individuals. The 6.7-kb deletion comprises of the first six of total seven exons and removes all of four Ig-like domains, leading to a truncated protein (Torkar et al., 2000; Wilson et al., 2000; Norman et al., 2003). Interestingly, the frequencies of *LILRA3* 6.7-kb deletion vary widely among ethnic groups, being much higher in Northeast Asians such as Japanese (71%), Chinese Han (76%), Chinese Manchu (79%), and Koreans (84%), compared to Europeans (15–26%), South Asians (10%), or Africans (7%) (Hirayasu et al., 2006; Hirayasu et al., 2008; Du et al., 2014). However, so far, the frequencies of the *LILRA3* 6.7-kb deletion have not been carefully investigated among the Han Chinese subpopulations across the geographic regions.

To date the function of LILRA3 remains obscure, but LILRA3 could bind to HLA class I molecules HLA-G and HLA-C (Jones et al., 2011; Ryu et al., 2011) and may act as an antagonist on other LILRs or a soluble ligand to other receptors (Torkar et al., 2000; Burshtyn and Morcos, 2016). In Caucasian populations, the *LILRA3* 6.7-kb deletion has been reported as a genetic risk for primary Sjogren’s syndrome (pSS) (Kabalak et al., 2009) and multiple sclerosis (MS) (Koch et al., 2005; Ordonez et al., 2009; Wisniewski et al., 2013; Ortiz et al., 2015; An et al., 2016). Nevertheless, our previous studies have demonstrated that, in Han Chinese population, the non-deleted (functional) *LILRA3* allele, rather than the 6.7-kb deleted *LILRA3*, was the genetic risk for pSS, systemic lupus erythematosus (SLE), and rheumatoid arthritis (RA) (Du et al., 2014; Du et al., 2015). A GWAS study has also reported the functional *LILRA3* was a risk factor for susceptibility to prostate cancer in Han population (Xu et al., 2012). These reports have provided strong evidence that the functional *LILRA3* is a genetic risk for multiple chronic diseases. However, whether the functional *LILRA3* is a novel susceptibility factor for AS has not been investigated. We undertook the present study (i) to investigate the frequencies of the *LILRA3*6.7-kb deletion among Han Chinese subpopulations across the geographic regions, (ii) to examine the possible genetic association between *LILRA3* and AS, and (iii) to examine whether *LILRA3* influences the disease activity in AS.

## Material and Methods

### Study Subjects

Two independent cohorts were enrolled, including 1,567 subjects (821 cases and 746 healthy controls) from Peking University Shenzhen Hospital (SZH) and 2,507 subjects (300 cases and 995 selected healthy subjects for case-control analysis by taking account of gender and age matching, and 2,207 healthy subjects for subpopulation stratification analysis, respectively) from Peking University People’s Hospital (PH). All patients with AS fulfilled the 1984 Modified New York Criteria for the diagnosis of AS (van der Linden et al., 1984). All cases and healthy controls are Han Chinese.

In the SZH cohort, the patients were recruited from the Department of Rheumatology of Shenzhen Hospital and from both out-patient and in-patient departments between Jan 2012 and May 2019. The healthy controls were from the Health Care Center affiliated to Shenzhen Hospital.

In the PH cohort, the patients were recruited from the Department of Rheumatology and Immunology of People’s Hospital and from in-patient department between Jan 2015 and May 2019. The healthy controls were recruited from the Health Care Center of PKU People’s Hospital, the First Affiliated Hospital of Anhui Medical University, and Gulou Hospital Affiliated to Medical College of Nanjing University, respectively (Du et al., 2014; Du et al., 2015). The baseline demographic characteristics of patients and controls are shown in **Table 1**. The geographic characteristics of the patients and controls from the two independent cohorts, according to the the Chinese administrative district divisions and the latitudes, are shown in the **Supplementary Table 4** and **5**, respectively.

**Table 1:** Demographic characteristics of the study cohorts.

Characteristic	SZH Han	PH Han	
No. of AS patients	821		300
No. of healthy controls	746	2,207^#^	995
Male (patients, %)	77.4		80.0
Male (controls, %)	76.5	24.6^#^	55.1
Age of patients*	31.7 ± 8.6		38.5 ± 15.4
Age of controls *	33.1 ± 9.0	37.7 ± 10.9^#^	39.9 ± 11.4
Disease duration*	8.7 ± 7.2		8.8 ± 8.1

AS, ankylosing spondylitis; SZH, Shenzhen Hospital; PH, People’s Hospital.

^#^the total healthy subjects from PH for subpopulation analysis.

*Mean ± SD years.

The study was approved by the Medical Ethics Committee of Peking University Shenzhen Hospital. Written informed consent was obtained from all participants.

### Subpopulation Stratifications

In SZH cohort, majority of the patients and healthy controls came from Guangdong, Hubei, Hunan, Jiangxi, Sichuan, and Fujian. In the PH cohort, majority of cases and healthy individuals were from Beijing, Tianjin, Hebei, Shandong, Liaoning, Inner Mongolia, Jilin, and Heilongjiang. In addition, a small proportion of healthy individuals in PH cohort were from Jiangsu and Anhui.

For the subpopulation stratification analysis, the two independent healthy cohorts were merged, and then, the total healthy subjects were classified into subgroups according to the Chinese administrative district divisions, i.e., Northeastern China, Northern China, Eastern China, Central China, Western China, and Southern China (He et al., 2017). For the case-control stratification analysis, the two independent case-control cohorts were respectively pooled, and the samples were then stratified into the three case-control sub-cohorts according to the latitudes, i.e., (i) latitude ≥ 35° (roughly corresponding to Northeastern and Northern regions), (ii) latitude 25–35° (roughly corresponding to Western, Central, and Eastern regions), and (iii) latitude ≤ 25° (roughly corresponding to Southern region). Of note, there were a number of cases and healthy controls from the metropolises of Shenzhen and Beijing, where the geographical location is no longer a good indicator of ancestral origin due to the impact of modern immigration (He et al., 2017). These individuals were self-identified their ancestral origins in the stratification analysis.

### Determination of LILRA3-del by Sequence-Specific Primer-Polymerase Chain Reaction (PCR-SSP)

Genotypes of the *LILRA3* 6.7-kb deletion polymorphism were obtained by PCR amplification with modified sequence-specific primers (PCR-SSP) (Du et al., 2014; Du et al., 2015). The cases and controls from the SZH cohort were genotyped at Shenzhen Hospital, and the AS cases from the PH cohort were genotyped at People’s Hospital. The genotyping success and confirmation rates were 99.1 and 100%, respectively. The genotyping dataset has been deposited in the figshare database (DOI: 10.6084/m9.figshare.9685619, https://figshare.com/s/0eab58f90f3b1b20a181).

In the PH cohort, the genotyping data for healthy controls were cited from our previous publications (Du et al., 2014; Du et al., 2015).

### Serum Dkk-1 Measurements

A total of 384 AS patients were measured for serum levels of Dkk-1. Serum samples were collected and stored immediately at −80°C prior to be used. All cases were genotyped for the *LILRA3* 6.7-kb deletion polymorphism. Quantification of serum Dkk-1 concentration was performed by using commercially available ELISA kits, according to the manufacturer’s instructions (R&D Systems, Minneapolis, MN). The detection range for Dkk-1 is from 31.3 to 2,000 pg/ml with an assay sensitivity around 15.6 pg/ml (catalogue number: DKK100).

### Power Analysis

The power analyses were performed retrospectively for the available samples (cases and controls), using a fixed minor allele frequency of 31.0% (the MAF in healthy controls from the combined cohort), a type I error *P* of 0.05, and an OR of 1.4. The PS software (version 3.0.14) was used for power calculation (available at http://www.mc.vanderbilt.edu/prevmed/ps).

### Statistical Analyses

The Hardy–Weinberg equilibrium (HWE) test was performed for the polymorphism, using Pearson’s goodness-of-fit chi-square test. The Pearson chi-square test was performed for the comparisons of allelic frequency differences between cases and controls. Odds ratios (OR) and 95% confidence intervals (CI) for genetic model analysis were calculated using logistic regression, adjusting for age and sex. The independent T-test was applied for analysis of serum levels of CRP, ESR, and Dkk-1, and the Bath Ankylosing Spondylitis Disease Activity Index (BASDAI) between two genotypic groups. All statistical analyses were conducted using program SPSS 16.0 (SPSS Inc., Chicago, IL, USA). The *P*-value < 0.05 was considered statistically significant.

## Results

The 6.7-kb deletion variant was in Hardy–Weinberg equilibrium (HWE) (*P* > 0.05) in healthy controls (data not shown). The study had a statistical power of 0.987 to detect the modest effect size of OR = 1.40, and a fixed minor allele frequency (MAF) of 31.0% (the MAF in healthy controls from the combined cohort) between LILRA3 and AS. However, the single-subpopulation study power was generally low (study power = 0.769 in Northern Han, 0.708 in Central Han, and 0.520 in Southern Han, respectively).

### Frequencies of *LILRA3* 6.7-kb Deletion Are Highly Differentiated Within Han Chinese Subpopulations

**Figure 1:**
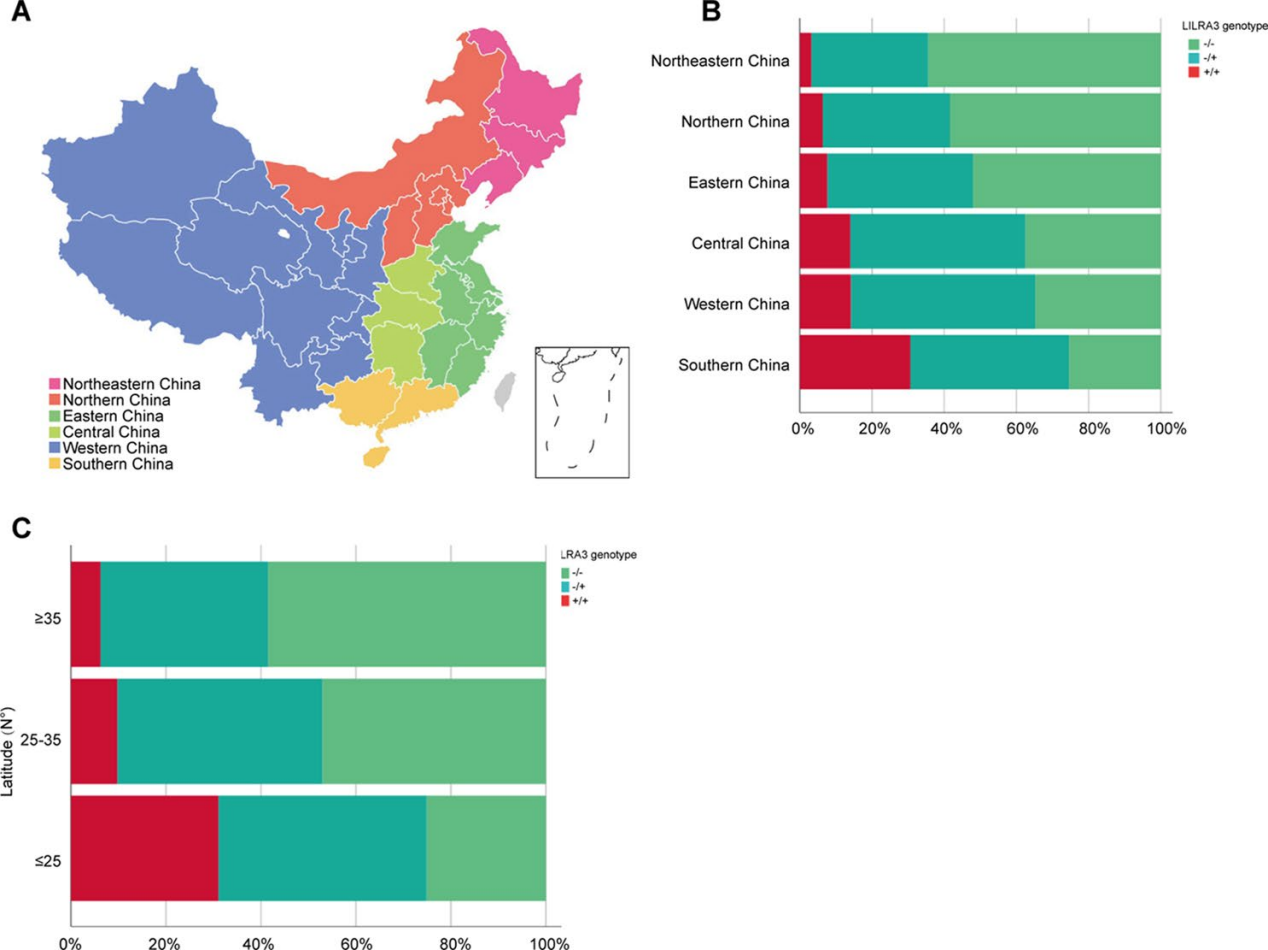
Geographical distribution of the *LILRA3* 6.7-kb deletion frequencies in the Han Chinese healthy individuals. **(A)** The combined healthy individuals cohorts were classified into subpopulations according to the Chinese administrative district divisions, i.e., Northeastern China (pink), Northern China (orange), Eastern China (green), Central China (light green), Western China (blue), and Southern China (yellow). **(B)** Genotypic frequencies of the *LILRA3* variations in the administrative geographic regions. **(C)** Genotypic frequencies of the LILRA3 variations according to the latitude.

As the frequencies of *LILRA3* 6.7-kb deletion were highly different in different populations worldwide and the study subjects were came from multiple geographical regions across China in present study, we hypothesize that the frequencies of *LILRA3* 6.7-kb deletion may also vary among Chinese Hans. To this end, we first pooled the two healthy cohorts and roughly classified the subjects into six subgroups according to the Chinese administrative district divisions (He et al., 2017) (**Figure 1A**). Interestingly, we found that allele frequencies of the 6.7-kb deletion varied remarkably according to the administrative district divisions. As shown in **Figure 1B** and **Table 2**, allele distribution of the 6.7-kb deletion were gradually decreased from Northeast (80.6%) to South (47.4%). Accordingly, frequencies of the functional *LILRA3* allele were gradually increased from Northeast (19.4%) to South (52.6%). Next, we investigated the geographical distribution of the *LILRA3* variants according to the latitude. As shown in **Figure 1C**, the homozygous of the functional *LILRA3* was about 4.5% at latitude ≥ 35°(roughly corresponding to Northeastern and Northern regions), 10.4% at latitude 25–35° (roughly corresponding to Western, Central, and Eastern regions), and up to 30.6% at latitude ≤ 25°(corresponding to Southern region). Collectively, the frequencies of *LILRA3* 6.7-kb deletion was highly differentiated within Han Chinese subpopulations. Frequencies of the functional *LILRA3* were reversely correlated with the latitude in Han Chinese, with the highest frequency seen in Southern Hans (52.6%).

**Table 2:** Geographical distribution of the *LILRA3* variations in Han Chinese healthy individuals, according to the Chinese administrative district divisions (n = 3,343).

Subpopulations	Latitude	Allele		Genotype		
		−	+	−/−	−/+	+/+
Northeastern (n = 31)	≥ 35(N°) (Northern Han)	50 (80.6)	12 (19.4)	20 (64.5)	10 (32.3)	1 (3.2)
Northern (n = 1,687)		2,565 (76.0)	809 (24.0)	985 (58.4)	595 (35.3)	107 (6.3)
Eastern (n = 682)	25–35(N°) (Central Han)	985 (72.2)	379 (27.8)	355 (52.1)	275 (40.3)	52 (7.6)
Western (n = 92)		283 (61.8)	175 (38.2)	86 (37.6)	111 (48.5)	32 (14)
Central (n = 229)		111 (60.3)	73 (39.7)	32 (34.8)	47 (51.1)	13 (14.1)
Southern (n = 232)	≤ 25(N°) (Southern Han)	220 (47.4)	244 (52.6)	59 (25.4)	102 (44)	71 (30.6)

N°, North latitude; (−), 6.7kb-deletion; (+), non-deletion.

### Functional *LILRA3* Seems to Be a Strong Genetic Risk for Development of AS, If the Study Subjects Were Not Geographically Stratified

**Figure 2:**
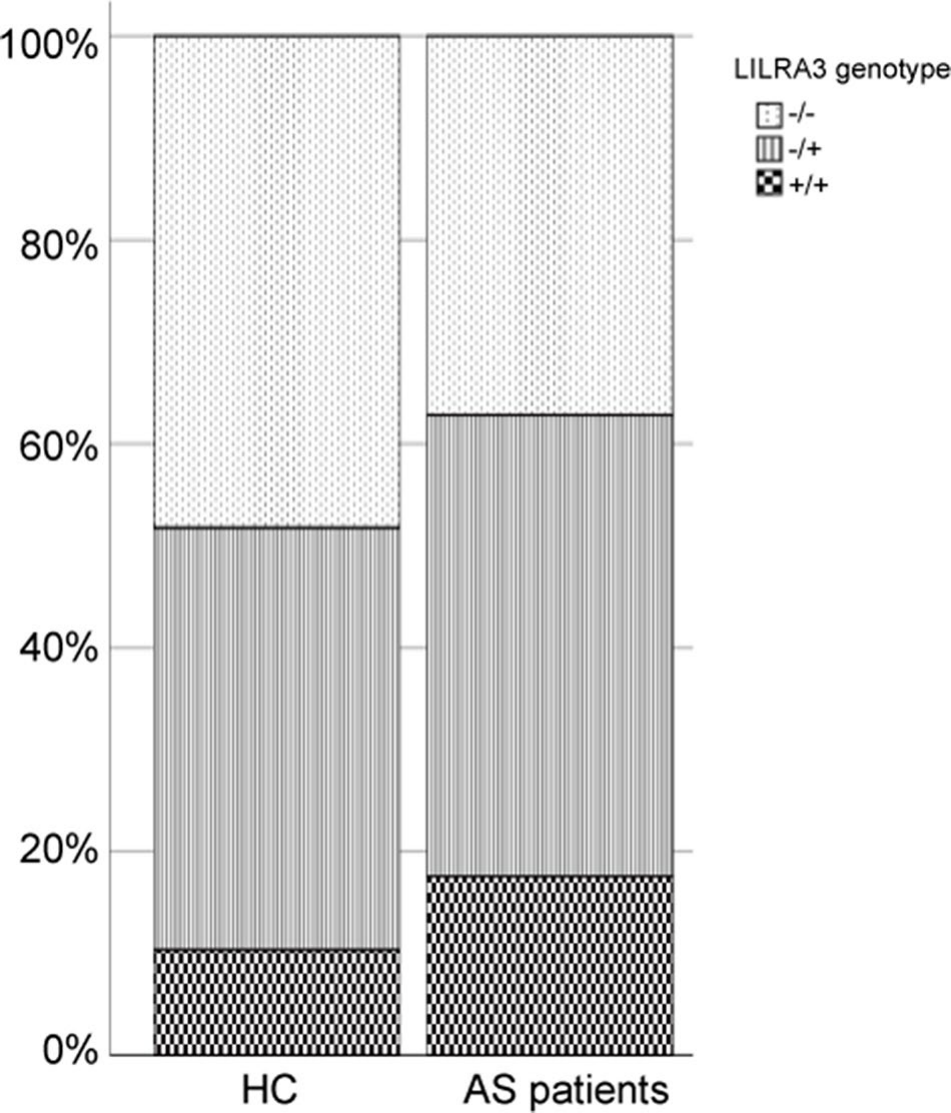
Comparison of the functional *LILRA3* frequencies between healthy controls and AS patients in whole study subjects without geographic stratification.

To investigate the possible genetic association between the functional *LILRA3* and AS, we first assessed the impact of *LILRA3* on AS susceptibility in whole study subjects. Interestingly, frequencies of the functional *LILRA3* were remarkably increased in AS patients compared with healthy controls, either in allele model (40.2% *vs.* 31.0%, *P* = 1.28´10^−12^, OR = 1.49, **Table 3**) or in almost all the alternative genotypic models (e.g., recessive model [+/+ *versus* -/- and +/-]: 17.6% *vs.* 10.3%, *P* = 1.17´10^−5^, OR = 1.66; **Figure 2** and **Table 3**). It seems that the functional LILRA3 is a strong genetic risk factor for AS susceptibility in Han Chinese, if the study subjects were not geographically stratified.

**Table 3:** Association analysis of *LILRA3* with AS in combined cohort, adjusting for sex and age.

	HC (n = 1,741)	AS (n = 1,121)	*P*-value	OR (95%CI)
***Allele model***				
−	2,401 (69.0)	1,341 (59.8)	1.28 × 10^−12^	1.49 (1.34–1.67)
+	1,081 (31.0)	901 (40.2)		
***Dominant model***				
−/−	840 (48.2)	417 (37.2)	2.83 × 10^−6^	1.46 (1.25−1.71)
−/+ and +/+	901 (51.8)	704 (62.8)		
***Recessive model***				
−/− and −/+	1,561 (89.7)	924 (82.4)	1.17 × 10^−5^	1.66 (1.32−2.07)
+/+	180 (10.3)	197 (17.6)		
***Co-dominant***				
−/−	840 (48.2)	417 (37.2)	3.42 × 10^−8^	1.37 (1.23–1.54)
−/+	721 (41.4)	507 (45.2)		
+/+	180 (10.3)	197 (17.6)		
***Over-dominant***				
−/− and +/+	1,020 (58.6)	507 (45.2)	0.098	1.14 (0.98–1.33)
−/+	721 (41.4)	614 (54.8)		

AS, ankylosing spondylitis; HC, healthy controls; OR (95% CI), odds ratio (95% confidence interval); (−), 6.7kb-deletion; (+), non-deletion.

### Stratification Analysis Reveals That Functional LILRA3 Is Associated With AS Susceptibility Only in North Han, but Not in Central and South Hans

**Figure 3:**
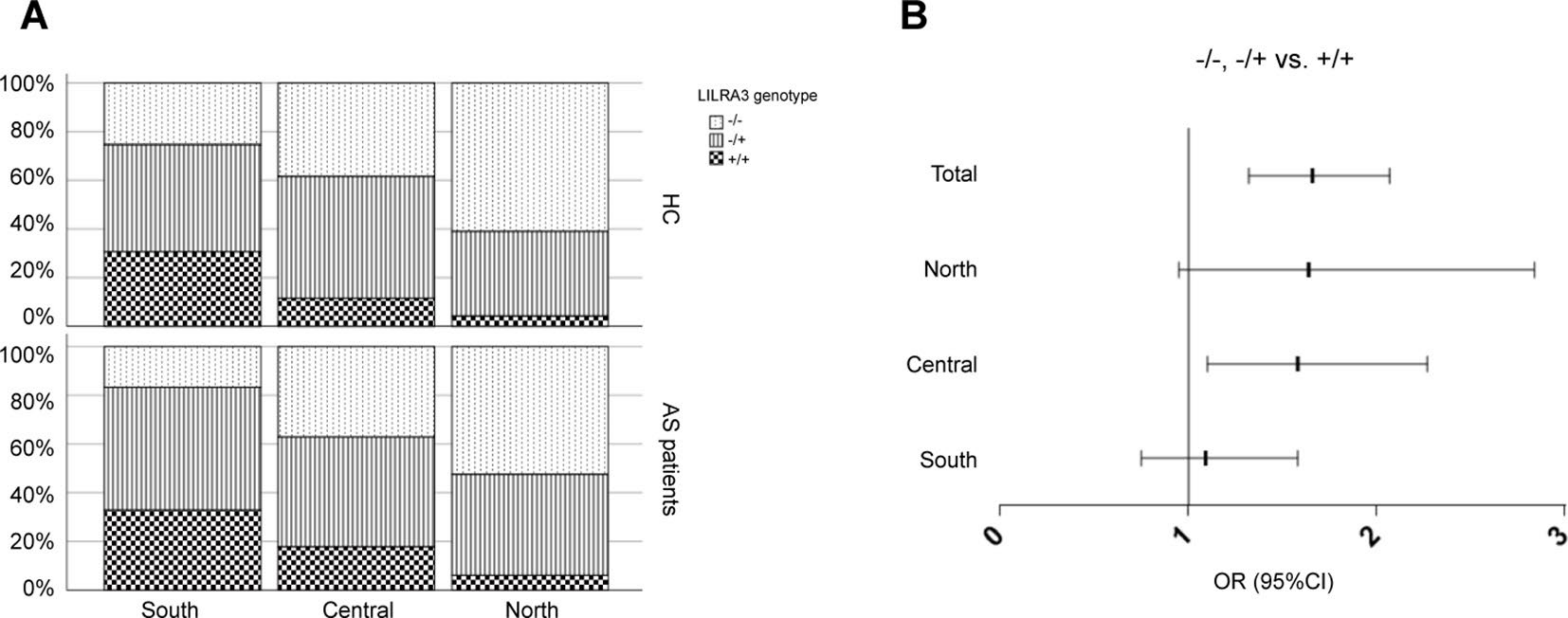
Comparison of the functional *LILRA3* frequencies between healthy controls and AS patients in Han Chinese subpopulations. **(A)** Either of healthy controls or AS patients were stratified into three subgroups, i.e., Southern Han, Central Han, and Northern Han, corresponding to the latitude ≥ 35, 25–35, and ≤ 25°, respectively. Frequencies of the functional *LILRA3* in each subgroup were compared between healthy controls and AS patients. **(B)** The forest plots of the association analysis between functional *LILRA3* and AS susceptibility with or without geographic stratifications (recessive model).

Previous studies have shown that the population stratification is a potential issue for genetic association studies and may confound results and cause spurious associations (Chen et al., 2009; Xu et al., 2009). As allele frequencies of *LILRA3* were highly differentiated in our healthy cohort, we next stratified the cases and healthy individuals into subgroups corresponding to the three ranges of latitude. The three subgroups were then renamed as: (i) Northern Han (corresponding to latitude ≥35°), (ii) Central Han (corresponding to latitude 25–35°), and (iii) Southern Han (corresponding to latitude ≤ 25°). As shown in **Figure 3A**, frequencies of the functional *LILRA3* (homozygous) were gradually increased in both healthy controls and AS patients from North to South. However, the associations between the functional *LILRA3* and AS susceptibility were different among the three subgroups. In the Northern Han subgroup, the functional *LILRA3* showed consistent association with AS susceptibility under almost all the alternative genetic models (allele model: *P* = 3.55 ´ 10^−3^, OR = 1.33; recessive model: *P* = 0.076, OR = 1.64; dominant model: *P* = 0.013, OR = 1.36; co-dominant model: 6.25 ´ 10^−3^, OR = 1.32; over-dominant model: 0.078, OR = 1.25), but not in Central and Southern Han subgroups (**Figure 3B**, **Tables 4** and **5** and **Supplementary Table 1**). Our results indicate that the functional *LILRA3* maybe a genetic risk for AS susceptibility in Northern Han subpopulation but not in Central and Southern Hans.

**Table 4:** Association analysis of *LILRA3* with AS, according to the latitudes and adjusting for sex and age (allele model).

	HC	AS	*P*-value	OR (95%CI)
***Allele model***				
Northern Han (≥ 35 N°)	n = 890	n = 412		
−	1,395 (78.4)	603 (73.2)	3.55 × 10^−3^	1.33 (1.1–1.61)
+	385 (21.6)	221 (26.8)		
				
Central Han (25–35 N°)	n = 619	n = 404		
−	786 (63.5)	482 (59.7)	0.081	1.18 (0.98–1.41)
+	452 (36.5)	326 (40.3)		
				
Southern Han (≤ 25N°)	n = 232	n = 305		
−	220 (47.4)	256 (42)	0.075	1.25 (0.98–1.59)
+	244 (52.6)	354 (58)		

AS, ankylosing spondylitis; HC, healthy controls; OR (95% CI), odds ratio (95% confidence interval); (−), 6.7kb-deletion; (+), non-deletion; N°, North latitude.

**Table 5:** Association analysis of *LILRA3* with AS, according to the latitudes and adjusting for sex and age (recessive model).

	HC	AS	*P*-value	OR (95%CI)
***Recessive model***				
Northern Han (*≥* 35 N°)	n = 890	n = 412		
−/− and −/+	852 (95.7)	387 (93.9)	0.076	1.64 (0.95–2.84)
+/+	38 (4.3)	25 (6.1)		
				
Central Han (25–35 N°)	n = 619	n = 404		
−/− and −/+	548 (88.5)	332 (82.2)	0.014	1.58 (1.10–2.27)
+/+	71 (11.5)	72 (17.8)		
				
Southern Han (*≤* 25N°)	n = 232	n = 305		
−/− and −/+	161 (69.4)	205 (67.2)	0.644	1.09 (0.75–1.58)
+/+	71 (30.6)	100 (32.8)		

AS, ankylosing spondylitis; HC, healthy controls; OR (95% CI), odds ratio (95% confidence interval); (−), 6.7kb-deletion; (+), non-deletion; N°, North latitude.

### Functional LILRA3 Confers an Increased Disease Activity in AS Patients

**Figure 4:**
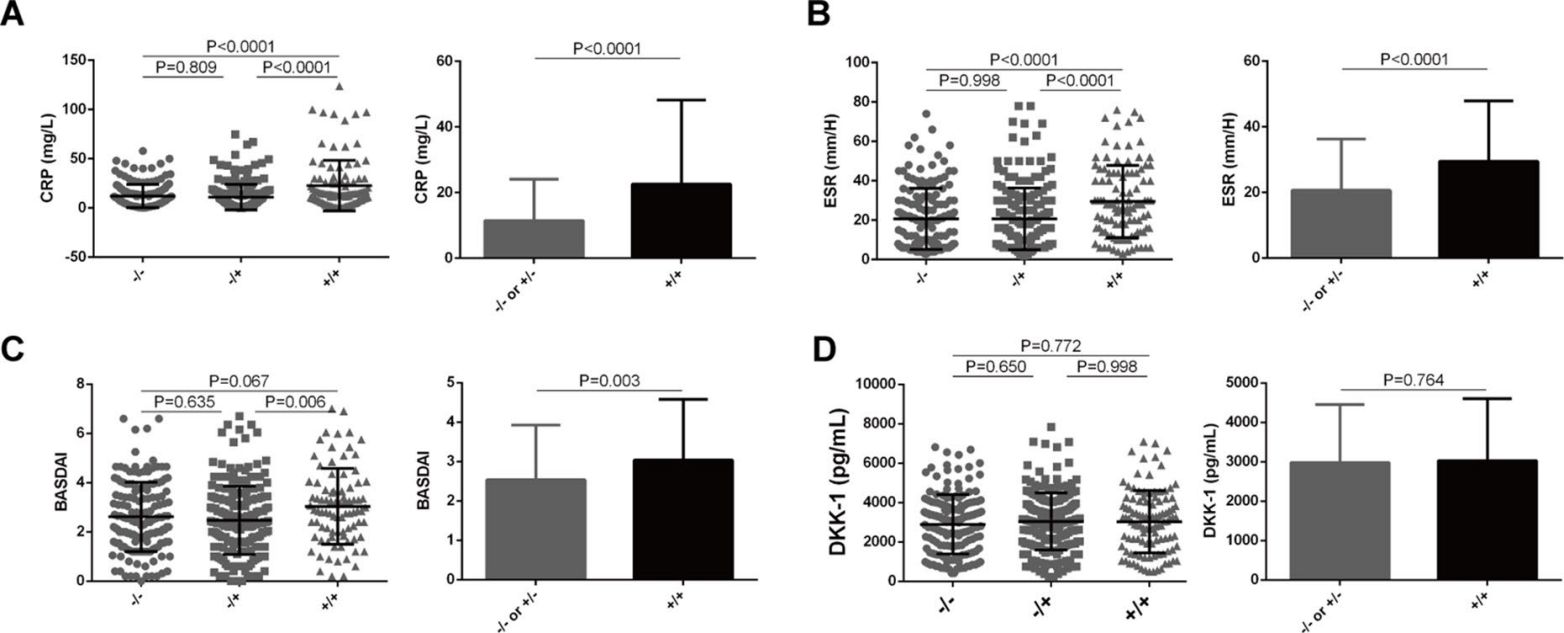
Association of *LILRA3* genotypes with disease activity in AS patients. The AS patients homozygous for the functional *LILRA3* had a significant higher levels of CRP, ESR, and BASDAI than nonfunctional *LILRA3* carriers **(A)**, **(B)**, and **(C)**, but not for Dkk-1 **(D)**.

We next examined whether the functional LILRA3 had an impact on disease activity in AS patients. As CRP and ESR are the two biomarkers most commonly utilized for evaluating AS disease activity, we evaluated the impact of *LILRA3* genotypes on serum levels of CRP and ESR in AS patients. As shown in **Figure 4**, the patients homozygous for the functional *LILRA3* had a significant higher levels of CRP and ESR than the nonfunctional *LILRA3* carriers (*P* < 0.0001 both for CRP and ESR, **Figures 4A, B**). Interestingly, we further observed that the BASDAI (a validated diagnostic test and gold standard for measuring disease activity in AS) was also significantly increased in AS patients homozygous for the functional LILRA3 (*P* = 0.003, **Figure 4C**). We also evaluated the impact of *LILRA3* genotypes on serum Dkk-1, a molecule related to AS disease activity. As shown in **Figure 4D**, no significant differences were observed for serum levels of Dkk-1 between different LILRA3 genotypes (*P* = 0.764).

## Discussion

Previous several studies have reported that frequencies of *LILRA3* 6.7-kb deletion vary widely across populations (Hirayasu et al., 2006; Hirayasu et al., 2008). Here, we demonstrate that allele frequencies of the 6.7-kb deletion also differ remarkably among the Han Chinese subpopulations across geographic regions, being the highest in Northeast China (80.6%) and the lowest in South China (47.4%), and positively correlated with the latitude. Conversely, frequencies of the functional *LILRA3* were reversely correlated with the latitude, being the highest in South (52.6%) and the lowest in Northeast (19.4%). If the study subjects were not carefully geographically stratified, the functional *LILRA3* seemed to be a strong susceptibility factor for AS. However, after stratifying the cases and healthy individuals according to the geographical regions, we find the functional *LILRA3* is mainly associated with AS susceptibility in North and Han, but not in Central and South Hans.

Genetic differentiations among Han Chinese subpopulations have been reported previously. Xu et al. (2009) reported that Chinese Han population is complicatedly substructured, with the main clusters corresponding roughly to Northern Han, Central Han, and Southern Han. By simulated case-control analysis, the study showed that the genetic differentiations among these clusters were sufficient to lead spurious associations in GWAS, if Han population was not properly stratified. Thus, any association studies should be carefully explained in Han Chinese population, especially when sample sources are diverse. Chen et al. (2009) reported that the structure of Han population is onedimensional and clearly characterized by a continuous genetic gradient along the north-south geographical axis, rather than the east-west pattern. Interestingly, the study further showed that the Cantonese is the most differentiated subpopulation from the Northern Hans. Our data are consistent with these findings; that is, the allele frequencies of *LILRA3* are mainly differentiated alongside the north-south gradient, being a much higher frequency in Southern Han. The mechanism for this gene selection within Han Chinese population is unknown. We speculate that the gene flow, environmental factors such as exposure to ultraviolet light, diet, life style, and immune systems suffering from different pressures might account for the differentiation between Han Chinese subpopulations.

Despite the well-established genetic association between *LILRA3* and autoimmune diseases, the molecular function of LILRA3 remains undefined. However, *LILRA3* is highly homologous to *LILRB1* and *LILRB2* in the extracellular domains, suggesting it may act as a soluble antagonist to these inhibitory receptors *via* shared ligands (Torkar et al., 2000; Burshtyn and Morcos, 2016). Previously, we and others have reported that the expression of *LILRA3* was significantly increased in RA and SLE patients. *LILRA3* had an impact on disease activity in RA and SLE (An et al., 2010; Du et al., 2015). Functional *LILRA3* conferred a risk to disease severity in RA patients with early disease (Du et al., 2014). Serum LILRA3 was one of the strongest independent markers for disease severity in patients with MS (An et al., 2016). In present study, we also find the functional *LILRA3* has an impact on disease activity in AS patients.

Dkk-1 is a key inhibitory molecule in the Wnt pathway and is critically important in bone homeostasis. Therefore, Dkk-1 may play an important role in AS pathogenesis (Heiland et al., 2012). Increased Dkk-1 levels have been linked to bone resorption, whereas decreased levels are linked to new bone formation (Li et al., 2006; MacDonald et al., 2007). However, there are inconsistent findings regarding the relationship between serum Dkk-1 levels and the occurrence of AS. For instance, several studies have reported that serum Dkk-1 levels were significantly increased in patients with AS compared with normal subjects or bone-related disease controls (Daoussis et al., 2010; Zhang et al., 2016). The inhibitory effect of Dkk-1 in sera from AS patients on Wnt pathway activation was negligible and may be functionally impaired (Daoussis et al., 2010). In present study, we didn’t find any differences between LILRA3 genotypes and Dkk-1 production.

One of limitations in current study is that the single-subpopulation study power was generally low due to the geographical stratification and the rare allele frequency of the functional *LILRA3* in Northern Han subgroup. Thus, it may lead to an increased chance of type II errors, i.e., a false negative result. Additional studies with larger sample sizes are desired to confirm our findings.

In summary, the present study provides the first evidence that the frequencies of *LILRA3* 6.7-kb deletion vary widely among the Chinese Hans across geographic regions and positively correlated to the latitude. The functional *LILRA3* is associated with AS susceptibility in North Han, but not in Central and South Han subpopulations. *LILRA3* has an impact on disease activity in AS patients. These findings suggest *LILRA3* is a common genetic risk for multiple autoimmune diseases and provide clues for further functional studies. Our study further highlights the importance of genetic differentiations among ethnicities, even within the subpopulations of an ethnic group.

## Data Availability

This manuscript contains previously unpublished data. The name of the repository and accession number are not available.

## Ethics Statement

This study was performed in accordance with the Declaration of Helsinki and approved by the Medical Ethics Committee, Peking University Shenzhen Hospital. All patients provided informed consent to participate in the study in accordance with the Declaration of Helsinki. The protocol was approved by the Medical Ethics Committee, Peking University Shenzhen Hospital.

## Author Contributions

HW contributed to the collection of DNA samples and clinical data from SZH cohort, participated in genotyping, ELISA experiments, statistical analysis and manuscript drafting. YW contributed to the collection of DNA samples from PH cohort, participated in genotyping, data analysis and manuscript drafting. YT, HY and XZ participated in the collection and interpretation of clinical data from PH cohort. GZ, YC and JL participated in the collection and interpretation of clinical data from SZH cohort. ZL participated in the study design and revised the manuscript. JG contributed to the study design and data interpretation, supervision of the data analysis, manuscript drafting and revision. QW participated in the study design, data interpretation, and manuscript revision. All authors read and approved the final manuscript.

## Funding

This work was supported in part by the National Natural Science Foundation of China (No. 31470875, No. 31670915, No. 31870913, No. 31711530023, No. 31530020, No.81871289, No. 81771678), Beijing Natural Science Foundation (No. 7162192), the Shenzhen Science and Technology Program for Basic Research (No. JCYJ20170307112009204), Traditional Chinese Medicine Bureau of Guangdong Province (No.20183011), and Sanming Project of Medicine in Shenzhen (No.SZSM201612009).

## Conflict of Interest Statement

The authors declare that the research was conducted in the absence of any commercial or financial relationships that could be construed as a potential conflict of interest.

## Abbreviations

AS, ankylosing spondylitis; LILRA3, leukocyte immunoglobulin-like receptor A3; MHC, major histocompatibility complex; GWAS, genome-wide association study; CRP, C-reactive protein; ESR, erythrocyte sedimentation rate; MS, multiple sclerosis; BASDAI, the Bath Ankylosing Spondylitis Disease Activity Index.
