# Supplementary material for: Frequencies of the LILRA3 6.7-kb Deletion Are Highly Differentiated Among Han Chinese Subpopulations and Involved in Ankylosing Spondylitis Predisposition
Source: Front Genet. 2019 Sep 18;10:869. doi: 10.3389/fgene.2019.00869 (PMC6760026; doi:10.3389/fgene.2019.00869)
Supplement: Supplementary file 1 [file Table_1.docx]

**Supplementary Table 1** Association analysis of *LILRA3* with AS, according to the latitudes and adjusting for sex and age (dominant model)

|  | **HC** | **AS** | ***P*-value** | **OR (95%CI)** |
| --- | --- | --- | --- | --- |
| ***Dominant model*** |  |  |  |  |
| Northern Han  (≥ 35 N°) | n = 890 | n = 412 |  |  |
| -/- | 543 (61.0) | 216 (52.4) | 0.013 | 1.36 (1.07-1.74) |
| -/+ and +/+ | 347 (39.0) | 196 (47.6) |  |  |
|  |  |  |  |  |
| Central Han  (25-35 N°) | n = 619 | n = 404 |  |  |
| -/- | 238 (38.4) | 150 (37.1) | 0.629 | 1.07 (0.82-1.39) |
| -/+ and +/+ | 381 (61.6) | 254 (62.9) |  |  |
|  |  |  |  |  |
| Southern Han  (≤ 25N°) | n = 232 | n = 305 |  |  |
| -/- | 59 (25.4) | 51 (16.7) | 0.020 | 1.66 (1.08-2.53) |
| -/+ and +/+ | 173 (74.6) | 254 (83.3) |  |  |

AS: ankylosing spondylitis; HC: healthy controls; OR (95% CI): odds ratio (95% confidence interval); (-): 6.7kb*-*deletion; (+): non-deletion, N°: North latitude.

**Supplementary Table 2** Association analysis of *LILRA3* with AS, according to the latitudes and adjusting for sex and age (co-dominant model)

|  | **HC** | **AS** | ***p*-value** | **OR (95%CI)** |
| --- | --- | --- | --- | --- |
| ***Co-dominant*** |  |  |  |  |
| Northern Han  (≥ 35 N°) | n = 890 | n = 412 |  |  |
| -/- | 543 (61.0) | 216 (52.4) | 6.25🞨10^-3^ | 1.32 (1.08-1.63) |
| -/+ | 309 (34.7) | 171 (41.5) |  |  |
| +/+ | 38 (4.3) | 25 (6.1) |  |  |
|  |  |  |  |  |
| Central Han  (25-35 N°) | n = 619 | n = 404 |  |  |
| -/- | 238 (38.4) | 150 (37.1) | 0.108 | 1.17 (0.97-1.41) |
| -/+ | 310 (50.1) | 182 (45.1) |  |  |
| +/+ | 71 (11.5) | 72 (17.8) |  |  |
|  |  |  |  |  |
| Southern Han  (≤ 25N°) | n = 232 | n = 305 |  |  |
| -/- | 59 (25.4) | 51 (16.7) | 0.105 | 1.22 (1.96-1.56) |
| -/+ | 102 (44.0) | 154 (50.5) |  |  |
| +/+ | 71 (30.6) | 100 (32.8) |  |  |

AS: ankylosing spondylitis; HC: healthy controls; OR (95% CI): odds ratio (95% confidence interval); (-): 6.7kb*-*deletion; (+): non-deletion; N°: North latitude.

**Supplementary Table 3** Association analysis of *LILRA3* with AS, according to the latitudes and adjusting for sex and age (over-dominant model)

|  | **HC** | **AS** | ***P*-value** | **OR (95%CI)** |
| --- | --- | --- | --- | --- |
| ***Over-dominant*** |  |  |  |  |
| Northern Han  (≥ 35 N°) | n = 890 | n = 412 |  |  |
| -/- and +/+ | 581 (65.3) | 241 (58.5) | 0.078 | 1.25 (0.98-1.6) |
| -/+ | 309 (34.7) | 171 (41.5) |  |  |
|  |  |  |  |  |
| Central Han  (25-35 N°) | n = 619 | n = 404 |  |  |
| -/- and +/+ | 309 (49.9) | 222 (55.0) | 0.207 | 0.85 (0.65-1.1) |
| -/+ | 310 (50.1) | 182 (45.0) |  |  |
|  |  |  |  |  |
| Southern Han  (≤ 25N°) | n = 232 | n = 305 |  |  |
| -/- and +/+ | 130 (56.0) | 151 (49.5) | 0.141 | 1.30 (0.92-1.84) |
| -/+ | 102 (44.0) | 154 (50.5) |  |  |

AS: ankylosing spondylitis; HC: healthy controls; OR (95% CI): odds ratio (95% confidence interval); (-): 6.7kb-deletion; (+): non-deletion; N°: North latitude.

**Supplementary Table 4** Geographic characteristics of two independent case-control cohorts and the healthy cohort from PH for subpopulation analysis, according to the the Chinese administrative district divisions

|  | **HC** | | | |  | **AS patients** | | |
| --- | --- | --- | --- | --- | --- | --- | --- | --- |
|  | PH | | SZH | Total |  | PH | SZH | Total |
| Northeastern China |  | 0 | 31 | 31 |  | 32 | 36 | 68 |
| Northern China | 1658^#^ | 744 | 29 | 773 |  | 171 | 14 | 185 |
| Eastern China | 549^#^ | 251 | 133 | 384 |  | 38 | 145 | 183 |
| Western China |  | 0 | 92 | 92 |  | 27 | 83 | 110 |
| Central China |  | 0 | 229 | 229 |  | 31 | 239 | 270 |
| Southern China |  | 0 | 232 | 232 |  | 1 | 304 | 305 |
| Total | 2207^#^ | 995 | 746 | 1741 |  | 300 | 821 | 1121 |

AS: ankylosing spondylitis; SZH: Shenzhen Hospital; PH: People's Hospital;

# the total healthy subjects from PH for subpopulation analysis

***** Mean ± SD years.

**Supplementary Table 5** Geographic characteristics of two independent case-control cohorts and the healthy cohort from PH for subpopulation analysis, according to the latitudes

|  | **HC** | | | | | |  | **AS patients** | | |
| --- | --- | --- | --- | --- | --- | --- | --- | --- | --- | --- |
|  | PH | | | | SZH | Total |  | PH | SZH | Total |
| North Han  (≥ 35 N°) | 1658^#^ | | | 744 | 146 | 890 |  | 255 | 157 | 412 |
| Central Han  (25-35 N°) | 549^#^ | | 251 | | 368 | 619 |  | 44 | 360 | 404 |
| South Han  (≤ 25N°) |  | 0 | | | 232 | 232 |  | 1 | 304 | 305 |
| Total | 2207^#^ | 995 | | | 746 | 1741 |  | 300 | 821 | 1121 |

AS: ankylosing spondylitis; SZH: Shenzhen Hospital; PH: People's Hospital

# the total healthy subjects from PH for subpopulation analysis

***** Mean ± SD years

N°: North latitude
